# An Investigation of Shelter Workers’ Perspectives on the Assessment and Management of Unowned Cat Welfare in the United Kingdom

**DOI:** 10.3390/ani16111641

**Published:** 2026-05-27

**Authors:** Avni Bhatia, Ana Maria Barcelos, Jenni L. McDonald, James Waterman, Lauren Finka

**Affiliations:** 1 Feline Welfare Research Team, Cats Protection, National Cat Centre, Haywards Heath RH17 7TT, UK; ana.barcelos@cats.org.uk (A.M.B.);; 2 RedPony Analytics, Caernarfon LL55 4EP, UK; james.waterman@cats.org.uk

**Keywords:** free-living, free-roaming, unowned, feral, stray, management, rescue, community, farm shelters, UK

## Abstract

Free-living unowned cats are prevalent across the UK and may experience numerous welfare challenges. However, there is limited research around this. Cat shelter workers were interviewed on the complexities of unowned cat welfare and management, current barriers and recommended solutions. A total of 25 participants partook in semi-structured one-to-one interviews, which were thematically analysed. Some of the main welfare concerns described were overpopulation, health issues, the absence of preventative medical care, cruelty, and neglect. Key barriers included limited resources, funding, personnel and infrastructure, alongside inappropriate management of cats not suited to domestic lifestyles. External barriers included insufficient government support, limited public awareness around cat welfare issues, and fragmented care provision of cats within the community. Other overlapping barriers include inconsistent terminology use and management practices within shelters and communities. Participants recommended more strategic use of shelter resources towards population management programmes including TNR, professional staff training, and the standardisation of terms such as “stray” and “feral”. They advocated for a shared responsibility framework for unowned cats involving animal welfare organisations, the veterinary sector, communities, and the government. This would include stronger collaboration between charities to share knowledge and establish industry best practice, alongside greater government support through the introduction and enforcement of legislation.

## Simple Summary

This study explored how cat shelter workers perceive the welfare and management of free-living, unowned cats within the UK. Through interviews with 25 shelter workers, major welfare concerns were highlighted including cat overpopulation, untreated health problems, lack of routine veterinary care, and cases of cruelty or neglect. Participants also described significant barriers to improving welfare. Internal barriers included limited funding, staffing shortages, and inadequate facilities, as well as difficulties caring for cats that are not suited to domestic living. External barriers included insufficient government support, limited public awareness of unowned cat welfare, inconsistent community care, and poor pet ownership practices. Confusion around terms such as “stray” and “feral,” and associated inconsistencies in management approaches, were perceived to further complicate effective interventions. Participants recommended more strategic use of shelter resources, particularly through expanding TNR programmes and targeted training for shelter workers. They also stressed the need for clearer, standardised terminology and stronger collaboration between charities to share knowledge and develop best practices. Better engagement with communities was seen as essential for raising awareness and promoting positive cat care practices. Finally, participants stressed the need for the government to introduce and enforce stronger legislation, supporting a shared responsibility for unowned cat welfare.

## 1. Introduction

Domestic cats are among the most popular companion animals, though many live in a liminal space between domestic and wild states [1]. They experience a wide range of relationships with humans, shaped by varying types of management and care provision [2,3]. Many cats may also move between living independently and under human care—being unowned, owned, or ‘semi-owned’—temporarily or permanently [4]. These transitions can follow any sequence and may be driven by the cat, but more often result from human actions such as abandonment, feeding, or rehoming efforts. Consequently, human behaviours and actions strongly impact cat populations and wellbeing. For instance, the abandonment or straying of unneutered owned cats increases the unowned population, highlighting the interconnectivity between these populations [5]. Homing kittens born to unowned free-living mothers can integrate them into the owned population. However, placing fearful or unsocialized adult cats in domestic settings may compromise their wellbeing and increase the risk of future relinquishment, straying, or abandonment [6,7]. Selective caregiving by humans can also foster dependency in unowned cats, leaving them vulnerable if support is withdrawn [1]. Since cat populations do not exist in isolation, meaningful long-term progress in welfare assessment and population management can only be achieved through a combination of approaches that address the root causes of overpopulation—preventing accidental litters through neutering while also reducing abandonment and straying through positive cat care practices [5].

The UK’s current urban population of unowned cats is estimated at 250,000 [8], with many more in rural areas, including farms [9]. These cats affect, and are affected by, their local environments and human communities. Many may experience poor welfare and quality of life due to their free-living lifestyle [10] and an absence of consistent human oversight, including limited access to preventive and timely veterinary care. Unowned free-living cats face multiple hazards including, but not limited to, road traffic, disease, parasites, human cruelty, and predation by canids and other wildlife. These risks can cause death, injury, chronic pain, trauma and stress [11,12,13,14]. Free-ranging cats, including unowned free-living and owned outdoor cats, may also affect local biodiversity through predation, disease transmission, resource competition, and displacement of native species [15,16], though such impacts are context-dependent and population-level evidence in the UK remains limited [17,18].

Internationally, strategies to manage unowned cat populations vary by regulatory context. For instance, the choice between lethal and non-lethal methods depends on regional factors such as ecosystem priorities, legal frameworks, and cultural or community attitudes towards cats [19]. In the UK, current animal welfare legislation protects all domestic cats, extending to stray and feral cats as animals commonly domesticated in the British Islands under the Animal Welfare Act 2006 [20,21]. However, the legal framework surrounding cats remains ill-defined in several respects; unlike other companion animals, cats are considered less domesticated as they can transit through a range of states—domestic, stray, semi-feral, and feral—which complicates the definition and application of ownership and welfare responsibilities [21]. Additionally, within the legislation, the terminology used to describe cats, such as ‘stray’ and ‘feral’, is not formally defined, which further adds to legal ambiguity (ibid). The Act also covers protection from both physical and mental suffering, though what constitutes mental harm is not explicitly detailed and is left to interpretation (ibid). The legal framework for cats also differs considerably from that of dogs in several respects; cat owners are not legally required to confine or control their animals, compulsory microchipping for cats came into effect nearly a decade after it was introduced for dogs [22] and no statutory duty exists for local authorities to manage stray or unowned cats [21]. Clear legislative or municipal responsibility for the assessment and management of unowned cat welfare therefore remains absent, with the practical burden falling on animal protection organisations in collaboration with veterinary practices [23], and individual community members. To add to that, the shelter sector is largely self-regulated via ADCH (Association of Dogs and Cats Homes) minimum standards which are not legally enforceable, with statutory regulation only firmly in place in Scotland yet [24].

TNR (trap–neuter–return/relocate), and its various adaptations (e.g., TNVR; trap-neuter-vaccinate-return), is the most common management strategy practised by animal welfare organisations and cat care professionals in the UK [5]. In addition to conducting TNR, UK shelter organisations play a significant role in temporarily housing and re-homing a substantial portion of the unowned cat population. However, some free-living cats come from backgrounds or genetic populations that limit their ability to adapt to domestic life with humans [25,26,27]. For these individuals, the shelter environment, often characterised by overcrowding [28], disease risk [29,30], and stress-inducing/exacerbating handling or housing practices [31,32] may compromise welfare [33,34]. Such cats may be housed briefly for essential neutering, vaccination, and parasite control before being returned to their original site or relocated to another free-living location. However, some may remain in shelters indefinitely under stressful conditions that do not meet their basic needs [35,36], leading to chronic stress and poor health [34,37]. These individuals are often harder to home, placing additional strain on already-limited shelter resources [5].

Terminology used to describe unowned cats is also largely subjective and unstandardised [38], complicating decisions about management at both population and individual levels. “Unowned cats” is typically used as an umbrella term covering categories such as ‘feral’, ‘stray’, ‘street’, ‘community’, ‘farm’, ‘homeless’, ‘free-living’, and ‘in- betweeners’ [3,26]. These labels may imply a range of independent or overlapping features including ownership history, assumed socialisation status, dependence on humans for resources, freedom to roam, a reference to their original environment [23,39], as well as whether the cat is perceived as a pet or a pest. However, these labels are often used inconsistently, with no standardised approach to assessment or categorisation [40]]. Although evidence is limited, such labelling may differentially impact the management approaches applied to individuals and their subsequent short- and long-term welfare experiences. For example, unowned human-fearful cats that do not behave aggressively could be more likely to be labelled as a ‘stray’ (i.e., previously an owned pet) rather than ‘feral’ or ‘inbetweener’. As a result, they may be admitted to shelters and homed as pets rather than managed via TNR [27]. However, not all ‘stray’ cats cope well in shelters [25], and the absence of human-directed aggression does not guarantee suitability for domestic life.

Unowned free-living cats occupy diverse lifestyle and social spectrums [3,41], shaped by local resources and socio-cultural perspectives on their management [24,42]. As such, a one-size-fits-all approach to intervention is unlikely to succeed. Research on the management and welfare of unowned cats in the UK remains limited. Insights from those working or volunteering with these cats at local levels can help identify key barriers to optimal assessment, decision making, management and welfare outcomes for these populations and support the development of targeted and effective solutions.

As a preliminary investigation, this study focused on collating the perspectives of cat carers within a single large UK cat welfare charity, enabling in-depth insights to be gained across a diverse stakeholder group but within a defined operational context. These findings aim to inform the development of future research frameworks that will explore these areas at a broader sector level.

Through semi-structured one-to-one interviews with stakeholders representing a diverse range of key roles integral to the care and management of UK unowned free-living cats, this study aimed to answer three key research questions:

1. What are the primary welfare concerns for unowned, free-living cats in the UK?
2. What are the challenges associated with current approaches to their welfare assessment and management?
3. What solutions and resources are needed to improve the assessment and management of unowned UK cats?

## 2. Materials and Methods

### 2.1. Ethical Considerations

The project received approval from the Human Ethical Review Committee at the University of Edinburgh (UoE)—Reference HERC_2024_053. All participants received a detailed information sheet outlining the purpose of the study and their rights. Participants were assured that personal data would be collected, processed, and stored in full compliance with the UK General Data Protection Regulation (UK GDPR) and the Data Protection Act 2018. It was explicitly stated that raw data would not be shared with any third party, including the charity for which participants worked or volunteered. The information sheet included a safeguarding clause to protect participant well-being. Participants were informed they could withdraw at any time before data collection ended. After withdrawal or completion, all original recordings and identifiable data were deleted, and transcripts anonymised and securely stored in password-protected files on a cloud-based server accessible only to the researcher (a UoE MSc student at the time of data collection).

A pilot study with 5 peers was conducted to evaluate the topic guide length, test the research design and questions, and identify potential problems. Feedback was sought on the logical flow and clarity of the questions to ensure they were easy to understand. Participants were also asked whether the session’s length and quality felt tiring.

### 2.2. Recruitment of Participants and Sample Size Estimation

The charity conducted an internal scoping process to identify a cohort of individuals who regularly interacted with cats across a range of roles, including both staff and volunteers, as well as those that occupied roles directly supportive of primary caregivers. This recruitment strategy ensured a diverse sample of stakeholders representing a range of experiences, roles, geographic locations, and demographic groups. To gauge interest, an advertisement was circulated among those identified. Interested individuals received a description of the study aims, an overview of the interview process, and a non-obligatory invitation to participate. Although the recruitment strategy aimed to capture a diverse range of demographic groups, the resulting sample was predominantly female. Data saturation guided the final sample size [43]. Data collection and analysis were concurrent: each interview was transcribed and coded to compare emerging themes with those from previous interviews. Recruitment continued until no new insights or themes emerged, indicating that saturation had been reached.

### 2.3. Data Collection

Semi-structured one-on-one interviews were conducted online via Microsoft Teams by AB, following a topic guide (Appendix A). The guide’s structure was informed by relevant global and UK-specific scientific literature on unowned cat welfare. The interviews were centred on the three research questions of the study (see Section 1) focused on welfare issues, practical management challenges, and future resource needs in the UK context. To support addressing these questions, the following topics were covered by the interviewer: the terminology used to describe unowned cats; key welfare concerns in the UK; current management strategies and welfare evaluation practices; and interactions with external stakeholders.

### 2.4. Data Analysis

Reflexive thematic analysis (RTA) was conducted within a constructionist epistemological framework to identify and explore prominent themes in the data. RTA is a qualitative method used to identify, analyse, and report patterns of meaning (themes) within a dataset. In a constructionist epistemological framework, it recognises that knowledge is not passively received but actively constructed by individuals within social and cultural contexts [44]. This approach highlights the researcher’s role in shaping analysis and interpretation, recognising that their perspectives and biases influence how themes are identified [45]. 

We followed Braun and Clarke’s [46] six-phase analytical process to conduct the RTA [47]. All interviews were transcribed verbatim in two stages: automated transcripts from Microsoft Teams were manually reviewed to ensure accuracy and increase familiarity with the data. Transcripts were anonymised before analysis, with personally identifiable information replaced by generic descriptors and each assigned a unique code (e.g., P1, P2).

Anonymised transcripts were imported into NVivo (Version 14) [48] for coding and theme generation. The process began with systematic coding, labelling text segments to capture key ideas. Codes were then clustered based on shared meanings or relationships (Appendix B). Once the codes were reviewed, an inductive analytical approach was used to identify patterns across the dataset and develop broader themes and sub-themes. To understand participants’ perspectives, we took a dual approach to the data: semantic analysis identified meanings directly expressed in the responses, while latent analysis explored underlying ideas, patterns, and assumptions not immediately visible in the surface text.

We practiced reflexivity (the process of examining one’s own thoughts, feelings, and biases) at all stages by

- Journaling thoughts post-interviews.
- Engaging in regular discussions with supervisors/colleagues to challenge assumptions.
- Examining how personal factors such as prior experiences, knowledge levels, preferences, and perspectives could shape data collection and interpretation.

## 3. Results and Discussion

The study involved one-hour long interviews with 25 stakeholders from a large UK cat welfare charity. Participants included staff and volunteers who regularly interacted with unowned cats through activities such as TNR, community outreach, shelter admissions, veterinary care and advice, rehoming, daily shelter care, welfare assessments, and behavioural support/advice.

Participant demographics:

The participants included 23 females and 2 males and were aged between 24 and 67 years (mean: 44.32; SD: 12.11, median: 41) and worked with unowned cats across urban, rural, and suburban regions in the UK. Their length of service within the organisation ranged from 1.5 to 45 years, with a median of 6 years (Appendix C).

Research themes:

From the coded data, six overarching themes were identified. Each theme included core components of the three overarching research questions and are outlined in Table 1. Individual themes are described and discussed in detail in the following sections.

### 3.1. Theme 1: Standardisation of Terms, Guidelines, Decision-Making and Processes

All interviewees raised concerns about the inconsistent terminology used to label unowned free-living cats, highlighting the need for standardisation and public education about the diverse lifestyle and sociality spectrums on which these cats exist. These findings align with previous research, which highlights ongoing ambiguity and misunderstanding around unowned cat classification and terminology as a key concern for care stakeholders [49].

*“I prefer the term ‘free-living’—it’s more respectful, acknowledging that they live independently, and we can offer support to them, even if that support isn’t always accepted.” (P13)*

*“Standardisation of a few terms with clear meanings that everyone understands would be preferable.” (P17)*

Participants reported that inconsistent and unclear terminology, combined with limited awareness of the individual needs of unowned cats, compromised their welfare. They felt that the use, and perceived misuse, of labels such as “stray”, “feral”, “community”, etc., affected how veterinarians, professional animal carers, and the public managed unowned cats. The term “feral” was described as the most frequently misused by members of the public, who they felt often struggled to distinguish between ‘feral’ cats, ‘strays’, and those with intermediate behaviours (i.e., ‘inbetweeners’). This misidentification was felt to result in inadequate care strategies being employed.

*“You’ve got this massive spectrum people don’t know about. Feral, semi-feral, inbetweener, farm cat, community cat—so many terms out there that aren’t quite understood and simply banded together.” (P12)*

Participants also felt that the term “feral” carried negative connotations in the public eye, which could distort perception and discourage interest in learning about the characteristics, lifestyle, and care needs of these cats. This aligns with existing literature which highlights that the usage of the term “feral” can, in certain contexts, devalue and depersonalise individual cats, othering them from the category of companion cats and rendering them less deserving of appropriate understanding and care [50].

*“Lay members of the public think that feral means aggressive or vicious, when in fact it means a wild animal that is not socialised to humans.” (P8)*

Some unowned cats, well-adapted to free-living lifestyles, may display ‘friendly’ but very context-specific interactions with human care providers. A common example includes cats labelled as ‘community cats’ that gather around feeding stations maintained by local residents [26,27]. These cats may tolerate human presence in order to access key resources such as food, but do not actively seek human social interaction for its intrinsic value [51]. Such observed ‘friendly’ behaviours may lead community members to label these cats as ‘sociable’ or ‘human-socialised’ ‘stray’ cats, and consider them suitable for homing, even when they may not be well suited to companion lifestyles [52,53]. Participants’ mentions illustrated these misconceptions clearly, for example:

*“Although some people might interpret that a cat is friendly because it lets you come close or even stroke it during feeding times, that doesn’t mean that the cat wants human company or is more suited to a domestic environment. It might just be better off as an outdoor cat and is only interested as you offer the resources it needs.” (P12)*

Gaining an understanding of, and prioritising, each unowned cat’s degree of human sociability at the individual level during management and decision-making processes may be particularly beneficial [41]. However, this approach is often overlooked when cat assessment and decision making rely predominantly on catch-all labels such as ‘pet’, ‘stray’ or ‘community’, rather than integrating more nuanced information based on individual cats’ historic and current relationship with humans and their current behavioural presentations.

For example, participants provided examples of how the often-binary approaches to cat labelling (i.e., friendly vs ‘feral’) and subsequent management made it hard to know how to meet the needs of cats that did not seem to clearly fit within a specific category. This was mentioned in the context of both cats originating from unowned free-roaming environments, as well as non-typical domestic environments such as hoarding.

*“We’re not clear within our literature […] explaining the difference between feral and friendly cats lacks nuance. It’s rare to find an absolute feral cat, especially in urban areas, as many have had some exposure to humans. This creates confusion for both the public and our staff. When we say a feral cat runs away from people, it’s easy to misinterpret that urban cats that don’t run away are not feral and can be brought into care. However, these cats are neither feral nor domesticated socialised cats fit to be in care. We need a gradient to describe different types of cats based on their environments […] Right now, we simply categorise them as either a feral or friendly stray.” (P19)*

*“Let’s pretend that I’ve found a cat that’s gotten into a nice routine of coming to my house everyday. It lets me feed it, but I can’t stroke it. Now I want to try and see what I can do to help this cat so I go to our organisation’s website, but nowhere can I find a description of the kind of cat I’m dealing with. So, it’s too black and white, we don’t talk about the rest of the spectrum. If it’s feral we TNR, and strayed pets would come into care and get rehomed; there’s not really a clear pathway for those cats that fall in the middle.” (P25)*

It is worth nothing that, as staff and volunteers, participants were likely already familiar with terminology adopted by the organisation, which may have influenced how they interpreted or reported word usage by the public [50]. However, as mentioned above, participants did not consistently use organisational terminology themselves, nor did they agree on shared meanings. An alternative pathway suggested by several participants was the development of basic behaviour and welfare assessment resources for shelter cat caregivers, such as checklists or flowcharts outlining key indicators of behaviour, health, and basic needs. One participant suggested that cat behaviour courses be made mandatory for all staff and volunteers.

*“We face challenges with varied terms, pronunciations, and levels of understanding. Standardisation of a few terms with clear meanings that everyone understands would be preferable. Additionally […] policies and clear decision-making processes involving relevant behavioural or veterinary teams should be established to prevent […] decisions that could compromise welfare.” (P17)*

In the absence of behaviour and welfare assessment resources [54], decisions about unowned free-living cats may be shaped by the subjective perspectives of stakeholders with varying levels of knowledge about feline lifestyle needs [55]. This can result in management strategies that fail to support optimal welfare, particularly for individuals admitted into shelters that may be better supported via alternative interventions [5]. The following quote illustrates how the lack of comprehensive guidance on decision-making clashes with carers’ subjective views and practices:

*“Our current policy for unowned and unsocialised cats is TNR only, and that they shouldn’t come into care. However, I know it still happens because this is a tricky one—people can have really different views and emotions about putting the cat back outdoors. We need to have more open conversations to tackle this.” (P3)*

Participants also emphasised the need to balance stringent processes and guideline adherence with flexibility to accommodate individual cats. While some degree of standardisation in labelling and categorisation was seen as important, participants suggested decision-making should remain adaptable to each cat’s specific circumstances.

*“When cats come from homes, we’ve got histories on them and can make decisions accordingly. With unowned cats, it’s difficult as there is no background. You need to get to know their personality and make decisions accordingly on a case-by-case basis. Not every cat needs a cosy home, and different cats benefit from different things.” (P2)*

Sector-wide collaboration could also enable resource and knowledge sharing, prompt revaluation of current practices, and identify areas for improvement or development [56]. For example, several participants proposed that by sharing expertise, organisations could collectively enhance the effectiveness of unowned cat management at a broader scale.

*“Collaborations are so important as there might be something that another organisation is much better at than us, or vice versa. I’ve been trying to kickstart a non-pet cat project that has led me into conversations with another organisation […] basically, they already have a database of homes that can take non-pet or in-betweener cats, but they haven’t always got the cats to fill those homes. Us being able to join up with them has meant that some of our cats have found suitable outlets through them. So, it’s a win for everyone—the cat gets out of care quicker, the adopter also gets a quick response, and we can continue caring for other cats in need.” (P11)*

This suggests that the responsibility for initiating and sustaining collaborations may often fall on motivated individuals or organisations with the capacity to take that step, rather than on any coordinated infrastructure built on shared knowledge, resources, and willingness. In the absence of a referral network or shared directory, many organisations may be unaware of what others could offer, or who to approach and how; this could lead to some charities operating in isolation, with initiatives shaped by trial and error, rather than by shared learning from the successes and failures of others [57]. Where organisations hold competing interests or conflicting values, divergent approaches may be adopted despite the shared overarching goal of improving cat welfare [56]. Consequently, cats and resources are not being matched efficiently, despite the capacity for this to exist. This also risks widening the gap between large and small organisations, which could be reduced through cooperative work, as suggested by a participant:

*“Sometimes when you go on social media, charities are sort of slagging each other off because the smaller charities are not happy that the larger ones have more resources and are not helping […] but they don’t know our situation and staffing issues either. So yeah, it’d be great if we all just work together because it should all be about helping cats at the end of the day.” (P15)*

Several participants suggested that collaborations should extend beyond shelters and welfare charities to include other cat care stakeholders such as veterinarians. For example, they proposed that veterinary curricula include dedicated training on pragmatic approaches to treating and managing unowned free-living cats.

*“Some vets just label everything as feral if it’s outside. They put it in a crush [cage] and don’t touch it until necessary.” (P25)*

*“Overexuberant vets want to treat conditions like cystitis in a feral cat, despite us advising that euthanasia might be more humane, as the cat would not fare well in containment for proper recovery.” (P13)*

There is evidence of the variation in veterinary perspectives on practices such as pre-pubertal neutering of unowned kittens [56]. Addressing these differences could involve conversations surrounding evolving scientific evidence, changing individual and community attitudes, and continuous professional development. This is essential to ensure that decisions regarding unowned cat welfare and management are not shaped by the sociodemographic differences amongst veterinarians but are instead applied consistently at the cat population level (ibid).

### 3.2. Theme 2: Cat Care Provision Within the Community and Community Outreach

Participants raised concerns about inconsistent or fragmented care provision and responsibility-taking for unowned cats within the community. They discussed the extent to which local residents were aware of unowned cats and their locations and whether residents were monitoring cat wellbeing and proactively reporting concerns or not.

*“With community cats, we rely heavily on the public to notify us if a cat appears injured or unwell. We’ll handle the trapping, vet visit, and return. The weakness is when no one reports an issue, due to indifference or fear of incurring vet bills.” (P23)*

When care was provided by community members, participants described it as often inconsistent or fragmented, with individuals rarely assuming full or long-term responsibility or ensuring cats received holistic care in addition to supplemental feeding. Financial issues were frequently cited as a contributing factor.

*“Feeding, loving, and giving them a name is not enough—comprehensive care requires addressing health needs, controlling the population through neutering, and ensuring safe living conditions.” (P17)*

*“I’m noticing that those caring for community cats are struggling now. It used to be easy to discuss and get people onboard with TNR earlier. Now people are panicking thinking, ‘I can’t afford to feed the cats’.” (P19)*

Participants highlighted gaps in public knowledge and varying perceptions of unowned cats’ health and care needs. These were thought to be linked to community resistance to neutering, failure to recognise when veterinary care is needed, poor pain management, and the provision of inappropriate food.

*“The general welfare issue is that they live on scraps. If they’re not fed an adequate diet, they won’t be strong enough to fight diseases.” (P4)*

*“Cats are good at masking their pain but people also think cats are solitary, independent creatures that don’t need as much help.” (P15)*

Participants reported that unowned cats often come to the attention of animal rescue and welfare organisations only after complaints about overpopulation, poor health, disease outbreaks, or escalating public nuisance, at which point interventions become more complex and resource intensive.

*“People don’t contact me when it’s one or two cats. They contact me when it’s 10 or 15 cats and that’s a much bigger problem to solve.” (P16)*

To this effect, the development of practical resources for the public to support effective information gathering and more timely interventions were suggested:

*“It would be useful to have a quick guide or checklist enlisting basic information and steps for the public. Often, concerned people who reach out lack the specific details we need, which delays getting the cat the help it needs. A simple first-step guide could help streamline this process and ensure we have the information required to act quickly.” (P18)*

Based on the participant perspectives, an ideal scenario would see all community members taking responsibility for local unowned free-living cats and their wellbeing, understanding their needs and knowing when and how to seek support from animal welfare stakeholders (e.g., charities and veterinarians). A more strategic and proactive approach incorporating human behaviour change is recommended. This includes raising awareness of how to identify early signs of poor health, reporting the presence of free-living populations before issues of overpopulation or human–animal conflict arise, and engaging with relevant organisations to support timely interventions such as neutering. Such approaches would target the root causes of welfare challenges rather than just their symptoms [58,59].

Public awareness of appropriate cat care provision and the impacts of human actions (or inactions) on cats’ lives is integral to their welfare. For example, selective caregiving, such as providing food or temporary shelter to free-living cats in the absence of taking responsibility for their neutering and vaccinations, has contributed to the growth of unowned cat populations [60,61], disease spread [10,62], and their reliance on humans, particularly in urban and semi-urban areas [63]. Participants’ perspectives echoed patterns in the broader literature [4,49], emphasising the need to raise community awareness about if, how and when to effectively intervene in the lives of unowned free-living cats to support positive welfare outcomes for them.

*“Education is key to improving the welfare of unowned cats and what people should or shouldn’t do if they come across these cats”. (P11)*

Participants also felt that public confusion around best practices in managing free-living unowned cats often stemmed from inconsistent approaches across animal welfare organisations. This confusion was seen as particularly evident in relation to potentially controversial issues, such as the euthanasia of FIV-positive cats from unowned free-living backgrounds. This is given that FIV may not necessarily be fatal or decrease longevity with appropriate veterinary support [64] and as such may motivate stakeholders to want to provide long-term care for these individuals [65].

However, participants pointed out that long-term shelter housing and/or homing of FIV positive cats to domestic lifestyles they are not suited to could compromise their welfare. At the same time, they stressed that not intervening (i.e., simply leaving these cats in situ) may also result in poor welfare outcomes.

*“Our charity has terrible press because we euthanise feral cats with FIV… but if not, then are you going to release a sick, poor animal back into the wild just to die a painful death?” (P20)*

Such issues can provoke emotionally charged debates and differing responses among cat care stakeholders, leading to ethical dilemmas and uncertainty about how to prioritise cats’ quality of life. Participants stressed the importance of transparent communication about the potential benefits of euthanasia for unowned free-living cats in certain circumstances, considering their overall quality of life. They also called for greater awareness raising and public education, beginning with the recognition that not all unowned cats require placement in domestic homes.

*“People think we’re being cruel for not taking the [feral] cat in and finding it a home when doing that would highly compromise its welfare.” (P23)*

*“Some people will try to socialise a feral cat—it’s like putting a tiger in a cage and trying to tickle its belly.” (P9)*

*“There’s a misperception that any cat can become a house cat.” (P13)*

All participants praised charity-driven community outreach and engagement programmes. These target high-deprivation areas to establish an on-ground presence, raise awareness, conduct neutering programmes, and empower local communities to provide more comprehensive and sustainable cat care. They suggested that greater collaboration with community leaders (e.g., mosque leaders) and local influencers in ethnically diverse areas could help build trust and further raise awareness of cat welfare initiatives.

*“I’d like to see education extending to everyday places people visit—like local corner shops, libraries, schools, and even groups like Brownies and Cubs. Imagine if hairdressers who often have lengthy conversations with clients, were given basic cat welfare information. They could impart that knowledge during their appointments to people in need. The idea is to have consistent messaging everywhere, not just targeting vets or pet shops. Everyone from local pubs to religious leaders could be involved, as different communities have different key contacts.” (P17)*

In line with participants’ recommendations, engaging/recruiting local influencers (e.g., religious leaders, shop owners, schoolteachers, hairdressers) as ambassadors to deliver key cat welfare information could strengthen community engagement. Given that even neighbouring regions may differ significantly in terms of animal welfare ethics [57], involving community members in the co-creation of interventions can help ensure that these are practical and tailored to local contexts, rather than based on assumptions or broad generalisations [66]. These individuals could encourage early reporting of cat-related issues, promote responsible caregiving behaviours, and build local support for ongoing management initiatives, helping to foster a more integrated and lasting impact.

Participants emphasised the importance of supporting individual community members who actively manage unowned free-living cat colonies, calling it a “social return on investment”. They noted that investing in these individuals can help address challenges that charities may be unable to manage alone due to limited resources.

*“As an organisation, we can’t help all the cats directly. However, if we support the people on the ground who are caring for these cats, we can extend our reach and ensure more cats receive the best possible care. Providing caretakers with tips and strategies for effective colony management would benefit everyone involved. We can offer practical advice on assessing cats and maintaining clean feeding stations to prevent community complaints.” (P19)*

*“I’ve seen people trying to trap cats in small rabbit traps. If we can teach caretakers to use the correct traps appropriately, they can help trap the cat, take it to the vet and make better decisions; so, they become the middle person. It’s about more people taking ownership of the unowned cat population; it needs to be a nationwide issue involving the whole community, rather than a charity’s problem. We need the public to help us and realise that not every cat can and needs to be rehomed.” (P21)*

Enhanced community engagement could also help to bridge specific charity resource gaps. Participants highlighted the shortage of suitable alternative outlets (i.e., non-pet homes) for cats deemed unsuited to domestic living alongside humans. Building databases of such options relies heavily on outreach to identify residents willing and able to offer such spaces, with greater community engagement potentially leading to greater access to suitable outlets for cats.

*“We still have a lot of old-school farmers that see cats as pests, but with minimal input, you can make them understand that the cats will be an asset to their farm.” (P12)*

This emphasis on working ‘with’, rather than ‘on’ stakeholders through community engagement has been widely echoed in the literature for improving animal welfare and management [66,67].

### 3.3. Theme 3: Cat Health and Vulnerability

Participants identified limited access to basic resources as a major welfare concern for unowned free-living cats. They emphasised the critical need for shelter and safety, noting that young kittens are particularly vulnerable to fox predation, while older cats may struggle with severe weather and risk hypothermia.

*“They don’t have a regular food source or a place to retreat to for shelter.” (P1)*

Participants unanimously identified the lack of neutering as the most pressing welfare concern for unowned free-living cats. They explained how a lack of neutering leads to increased health risks and contributes to overpopulation, resulting in more at-risk cats competing for limited resources. Unneutered cats were described as more vulnerable to health issues linked to fighting and mating (e.g., cat bite abscesses) and disease transmission (e.g., FeLV, FIV). Respondents also felt that unowned cats were generally more susceptible to disease and parasite infestations due to missing out on preventative treatments such as vaccinations and worming.

*“The primary welfare concern is unchecked breeding. The Queen is worn out because of continuous pregnancies and litter rearing. The unneutered Toms are getting into fights. We’ve also seen litters where every feral kitten had something wrong with its eyes due to diseases.” (P4)*

A recurring concern among participants was the combination of overpopulation and limited access to preventative medical care, particularly in urban areas.

*“City cats mix and interact with other colonies, so the lack of vaccinations often leads to the spread of diseases.” (P20)*

*“From what I’ve seen, dental disease can be the worst for a cat that’s on its own, affecting its ability to eat and overall health”. (P22)*

In rural areas, unowned free-living cats may be perceived as beneficial due to their symbiotic relationships with farmers, for example, receiving shelter and basic care in exchange for rodent control [1]. However, participants noted that such relationships are less common in urban environments, where cats may still rely on degrees of human support but are not expected to serve functional roles for humans and face greater risks from resource competition, disease transmission, and human–animal conflict. This is corroborated in previous studies, which found that cats in urban environments were significantly more exposed to zoonotic pathogens than those in peri-urban or rural areas [68], and FIV seroprevalence was much higher urban strays [69].

*“We need to recognise the different needs of different types of cats, depending on their environments. Unowned cats in urban areas may fit into overlapping categories due to a higher degree of socialisation, as even feral cats might have some human contact, and disease transmission in such dense areas is much quicker. This differs from rural communities, where cats are more self-sufficient and reliant on wildlife.” (P16)*

This highlights the importance of understanding the distinct challenges faced by different populations of unowned cats, and designing targeted interventions based on their demographics and the geographical and societal contexts in which they live [5]. Participants noted that overpopulation hinders the practical and ethical management of unowned cats. When the number of individuals requiring TNR or veterinary care exceeds available resources, delays in treatment can worsen welfare outcomes. Larger populations also pose logistical challenges, particularly in coordinating cat trapping with available veterinary appointments. This can lead to extended cattery stays for cats unaccustomed to confinement or human handling, further compromising their wellbeing.

*“It’s difficult to get a vet for an open-ended appointment when aiming to trap a cat. If there’s no neutering appointment available when the cat is trapped, it stays confined longer than ideal, impacting its welfare.” (P11)*

While some health risks for unowned free-living cats are circumstantial, others are directly caused by humans. Participants recounted members of the public threatening to harm cats if they refused to help them. They also described community members sharing that cats had been stoned, shot, poisoned, or drowned.

*“Some people are not nice to stray cats… they will throw stones or hose them with water.” (P14)*

*“If they are threatening to shoot or poison the cats, that’s when we would relocate them.” (P23)*

A recent forensic study from Spain, examining 53 cats drawn predominantly from stray feline colonies, found that more than half had died from non-natural causes such as blunt force trauma, poisoning, and projectile injuries, corroborating our participants’ accounts of stray cats being especially vulnerable to deliberate abuse [70]. Comparable research in the UK remains limited and given that deliberate harm represents a significant welfare concern for unowned cats [71], similar investigation could prove valuable in both quantifying the scale of the issue and raising broader public awareness and sensitivity around cat welfare.

Participants also mentioned public reluctance to tolerate the return of unowned cats to their capture sites following neutering.

*“TNR gets difficult because people often want the T and the N, but not the R; they see the cats as a nuisance and want them gone.” (P8)*

### 3.4. Theme 4: Cat Ownership

#### 3.4.1. Role of Cat Owners

Stakeholders consistently emphasised the link between pet ownership practices and the prevalence of unowned cats. Unneutered owned cats were identified as a key contributor to the unowned population and poor cat welfare outcomes, through abandonment and accidental litters.

*“Many people don’t want to give up their cats but struggle with the cost of living because veterinary bills are ridiculous […] It’s leading to more sick, unchipped and unneutered abandoned animals.” (P20)*

*“Sometimes people get a kitten and then realise, as it grows up, that it’s not cute and fluffy anymore but actually quite a lot of work. So, they throw it out, and it ends up getting beaten up by the local cats, getting pregnant, or turning up a few streets away or on someone’s doorstep. Other times, people deliberately let their cats out, thinking they’ll breed, and they can make some money from selling the kittens. But that comes with major welfare risks—the cat might be too young to get pregnant, could have a very difficult pregnancy, and there’s also the risk of catching diseases like FIV.” (P6)*

Given that many cat owners in the UK allow their cats outdoor access [72], their decisions and capacity to neuter, microchip, and vaccinate can influence the wider cat population, as the boundary between owned and unowned cats is permeable [73]. Providing owners with knowledge about cat behaviour, needs, and the costs associated with all stages of ownership [5] may help reduce the risk of abandonment [74]. Participants strongly emphasised the importance of educating and supporting cat owners in relation to neutering.

*“Animal welfare and responsible pet ownership should be compulsory subjects in primary schools so kids can go on to teach their families at home.” (P20)*

*“I believe there need to be more media campaigns and messaging around—‘why is it so important to neuter your cat?’[…]” (P3)*

*“We used to have what we called ‘spay days,’ where we’d go out to the vets, give people information about neutering, write out vouchers, and leaflet the area […] we ended up learning a lot about the unowned cat population too, because people would often come and talk […] about things like stray cat colonies in their area or other unneutered cats they knew of.” (P4)*

Animal health and welfare professionals need to work with the knowledge that animals are interconnected with communities, which are part of larger social systems. Individuals relinquishing animals for financial or housing reasons are more likely to be from marginalised groups, experiencing systemic and structural barriers to wellbeing and access [75]. This illustrates how sociodemographic and policy factors interact to influence individuals’ capacity and willingness to care for their pets, and, in turn, shape unowned cat welfare [56]. Therefore, without upstream prevention, i.e., supporting owned cats and their owners, any downstream interventions for unowned cats will remain reactive and insufficient.

#### 3.4.2. Government Support

Participants felt that while charities play a crucial role in educating and supporting cat owners, government involvement is essential to establish a coordinated framework that enhances the impact of these initiatives. It was suggested that government support could help address persistent challenges in cat ownership and care provision.

*“There need to be stronger laws around cat ownership, because no matter how hard we try to change human behaviour, sometimes people will only change if they’re governed to do so.” (P9)*

*“We need the government to make legislative changes around cat ownership and breeding. Without that, we’re just firefighting, and it’s taking a toll on both us and the vet industry, with so many people burnt out from compassion fatigue.” (P6)*

*“If the government made it mandatory that anyone owning a cat had to have a license, neuter and microchip their cats, I don’t think we’d have such an issue with unowned cats. Also, if we could educate communities across the UK to undertake TNR through a government-backed initiative […] such mass neutering would massively solve the problem of overpopulation.” (P9)*

Abandonment may stem from a range of factors, from circumstantial inability to provide care to wilful disengagement from ownership responsibilities, compounded by broader socioeconomic and systemic challenges including a lack of pet-friendly accommodations, rising veterinary costs, and community attitudes towards cat ownership [76]. Previous research indicates that free-roaming cats are more prevalent in socioeconomically disadvantaged communities [56,77]. Greater government involvement could be especially beneficial where abandonment is driven by social, political, and economic factors, thereby reducing the burden currently carried by charities and ensuring that support still exists for those who may face barriers in accessing charitable provisions [76].

Participants praised the introduction of mandatory microchipping legislation for pet cats in England as a positive step toward reducing the number of lost or abandoned pets that may contribute to the unowned cat population. Participants based outside England expressed hope for similar legislation to be adopted across the wider UK.

*“I’m desperately hoping that the mandatory microchipping laws will bring positive changes since a third of the cats coming into care are strays and I suspect many of those are just missing pets. It would allow us to scan and reunite these cats with their families much quicker, shifting focus to managing and caring for other cats in need.” (P17)*

However, participants also expressed concerns about enforcement, particularly among financially constrained owners. Although the new law mandates fines for non-compliance, it exempts farm, community, and feral cats from compulsory microchipping [22]. Participants warned that rising living costs may lead some unregistered owners, unable to afford microchipping, to abandon their cats.

*“The microchipping law is great, but who’s going to be policing it and how? I mean, if people are already facing financial trouble to get their cats neutered, then getting them microchipped will be just another thing that they’re not going to be able to do. What if they get scared and abandon their cat?” (P4)*

While some charities offer discounted microchipping schemes, access depends on individual pet owners seeking out support. Based on participant insights, government involvement in targeting cat owners with greater need for practical support with cat care, facilitating direct access to subsidised microchipping, and implementing targeted educational campaigns could facilitate better cat care practices for both owned and unowned cats; this may improve compliance and reduce disproportionate impacts on economically disadvantaged groups.

Despite optimism that compulsory microchipping legislation will facilitate positive change and ease pressures on frontline services, recent data suggest uptake has plateaued: an estimated 2.6 million cats remain unchipped, and around 700,000 have outdated registration details, rendering their microchip useless [78]. This may reflect a combination of owner ignorance and rising financial pressures related to living costs and veterinary care [5]. Research suggests that punitive and enforcement-led policies disproportionately burden vulnerable populations and fail to address the root socioeconomic causes of non-compliance. Counterintuitively, such approaches have been linked to negative impacts on both human well-being and cat welfare [62]. International findings indicate that integrated approaches combining financial and resource support, community engagement, and evidence-based policy yield more sustainable and humane outcomes (ibid).

Drawing on participant perspectives and the wider literature, a shared responsibility framework involving government, veterinarians, animal welfare organisations, and communities could be explored to strengthen the assessment and management of unowned cat welfare in the UK. For example, in Italy, management guidelines for unowned cats are legally established and fully publicly funded, with municipalities, veterinary services, and animal welfare organisations all sharing responsibility. Initiatives within their framework include the registering of cat colonies and their caretakers and regulated neutering programmes [79].

### 3.5. Theme 5: Resource Allocation and Management

#### 3.5.1. Financial and Human Resources

Several participants reported that the nature of resource availability and its distribution had led to ad hoc, uncoordinated approaches at local levels. This was seen to hinder targeted approaches and efficient resource use in managing unowned cats. All respondents agreed that greater financial, human, and physical resources were needed, given the perceived scale of the problem.

*“I think the solutions we have are pretty good, but we just don’t have the resources: the space, the funds and the staff to deal with the extent of the problem. But then it’s hard to determine, you know—you could double what you have at the moment, and it still might not be enough.” (P1)*

This reiterates the need for greater cross-sector collaboration for more efficient matching of resources where capacity is limited.

A recurring sentiment was the need for more personnel, which participants felt could be addressed through stronger external promotion of volunteering opportunities to support recruitment.

*“Before I got in touch with the charity, I assumed the only thing you could do as a volunteer was fostering cats. That’s far from true as there are many roles requiring different skills.” (P16)*

Volunteers can be a meaningful operational asset in this sector where resources are otherwise limited [76], and organisations may be able to achieve more provided they enable volunteers efficiently to maximise output and benefits. A study by Spehar and Wolf [80] corroborates that ‘citizen science’ and volunteer contribution can fill critical gaps in free-roaming cat management, showing how the efforts of a single volunteer produced data and outcomes that professional bodies were not generating (ibid).

One participant highlighted a perceived lack of diversity within both the organisation and the wider sector, suggesting this limits opportunities to engage with diverse cultural perspectives and practices. The potential benefits were described as expanding the foundation of lived experience available to inform cat management practices across different communities and locations, helping to identify which approaches have been effective or ineffective in the past.

*“You look around and it’s all similar people practically from the same background and experiences. You don’t hear other people who say, ‘Well, in my country, this is how we manage unowned cats’. It doesn’t give you different perspectives to learn from or try.” (P13)*

This perspective is echoed in the literature, as a recent study [81] underscores both the power and the responsibility of institutions to make changes that enable more cultural knowledge and lived experiences to influence animal welfare management (ibid).

Given the emotionally and physically demanding nature of TNR work, participants expressed the importance of dedicated TNR teams for the long-term management of unowned free-living cat populations. The complexity and unpredictability of cat trapping, combined with the risk of compassion fatigue, were cited as key barriers. Participants unanimously supported the need for more intensive neutering programmes targeting both owned and unowned cats, alongside full-time TNR teams to provide support.

*“I would love to see a […] TNR team as it’s really a skill and the process can be so all consuming. I think we expect a lot out of our TNR volunteers.” (P7)*

Several participants also expressed concern that de-prioritisation of initiatives targeted towards unowned cats may exacerbate existing welfare and management challenges, particularly if unowned free-living cats lack advocates to ensure they receive appropriate support.

*“I feel that relinquished cats and kittens get prioritised and their welfare gets looked after better—probably because they are easier to handle and look at on case-by-case basis. Whereas for unknown, unowned cats with no history, the welfare standards and management approaches are not consistent enough […] across the organisation.” (P20)*

*“It would be ideal to have a team of trained people whose sole job was to deal with all cases of unowned cats only.” (P14)*

However, resource allocation and management can become contentious as finite resources may require difficult decisions and a careful balance between reactive versus proactive strategies and individual cat versus population-level benefits [5]. For example, while the deprioritisation of extensive TNR programmes for unowned cats may limit the means to appropriately support on all individual cases, investing in the targeted neutering of owned cat populations may be most effective in stemming the flow of cats entering into the unowned population in the first place (ibid). This highlights that while all stakeholders agree that interventions for unowned cats should be more proactive in nature, with charity resource constraints, the most effective routes to achieve the desired outcomes of unowned population reduction and improved welfare may require pragmatic approaches and decision making to make the best use of finite resources.

#### 3.5.2. Alternative Solutions and Specialised Care

Participants noted a shortage of non-pet or ‘alternative’ outlets (e.g., stables, farms, outdoor homes), for cats with specific lifestyle needs. They recommended proactively developing a comprehensive database of such homes and fosterers, alongside enhanced support for adopters, including detailed follow-ups to assess the success of homing outcomes.

*“It would be beneficial to have detailed follow-up documentation to support adopters that take in in-betweeners or cats requiring special care, ensuring they are well-prepared for the unique challenges they might face.” (P11)*

This is also reflected in the literature as there is limited guidance on determining the socialisation threshold that should inform placement decisions, and even where adoptive homes for poorly socialised cats are being found, the impact on both cats and adopters remains unclear [53]. No studies have evaluated post-adoption behaviour and well-being of such cats, or adopters’ experiences and satisfaction with these placements (ibid). One study does suggest, however, that adopters who received counselling about the spectrum of cat–human socialisation at the time of adoption reported fewer concerns afterwards, indicating that shelters could play a meaningful role in preparing adopters for the acclimitisation period [82]. This could not only ease pressure on already-limited shelter resources by redirecting the care of special needs cats through alternative placements, but also lower the risk of relinquishment, thereby preventing future strain on charity resources.

### 3.6. Theme 6: Research, Follow-Up and Continuous Improvement

The interviews highlighted a critical need for context-specific research and innovative approaches to improve the management and welfare of free-living unowned cats. This included optimising welfare within TNR programmes and evaluating the welfare impacts of TNR versus potentially less-suitable management pathways for unsocialsed cats (e.g., shelter admission and homing, particularly of ‘feral’ kittens). Investigations into the impact of homing FIV-positive ‘stray’ cats to domestic indoor environments and their ability to cope were also mentioned. Another participant highlighted the difficulties handlers face during the TNR process, and the need for research into improved strategies and alternatives that ensure ongoing support for cats beyond the immediate TNR intervention.

*“If we could speak openly about it, maybe we could come up with some research investigating better ways of catching some of these animals, probably using drugs, that are so difficult to catch but need to be caught urgently. I think there should be a lot more research done about feral cats and how we can support them beyond TNR.” (P13)*

Another participant expressed the need for ongoing evaluation of current policies and practices to ensure alignment with the latest evidence-based research and support continuous improvement.

*“One of our weaknesses is that we don’t do any follow-up, which I would like to see built into our management practices. It would be fantastic to follow up, create case studies, and combine them with research to help inform future policies and guidance. We need a continuous assessment of our interventions so that we’re not just doing what we’ve always done; we can see whether it’s having an impact, but also if it’s the right welfare thing to do, because if it’s not then it’d be good to amend that and move as time and knowledge progresses.” (P17)*

More emphasis on research, follow-up, and evidence-based guidelines for the issues raised by participants could reduce variability in cat welfare and management, while making practice more transparent and replicable [83]. This aligns with the wider behavioural-science literature, which suggests that standardised outcome measures are needed to identify which components of an intervention are effective, track change over time, and compare approaches across settings [84]. However, without standardisation, monitoring progress becomes difficult, particularly when multiple stakeholders are involved [84]. Clear operational definitions and appropriate training are therefore important to improve consistency and inter-rater reliability in the evaluated measures being reported (ibid). This is especially relevant for free-living, unowned cat populations, where routine follow-up is often difficult and outcomes are harder to assess over time.

## 4. Conclusions

The following section provides a synthesised overview of findings from the six main themes, framed around our key research questions:

1. What are the primary welfare concerns for unowned, free-living cats in the UK?

The primary welfare concerns identified by stakeholders in this study fall into two broad categories. First, concerns arising from the immediate circumstances of unowned cats, such as limited access to preventative health checks, high disease prevalence, and exposure to extreme weather. Second, concerns stemming from human action or inaction, including misidentification or mislabeling of cats leading to inappropriate management, abuse, inconsistent or inadequate care, unsuitable rehabilitation efforts, and fragmented responsibility across animal welfare organisations, veterinary services, and the public.

These human-influenced issues align with those identified by Rioja-Lang et al. [71], who found, based on expert opinion, that the main welfare challenges for both owned and unowned cats in the UK stem from limited understanding of feline welfare needs among care stakeholders, community members, and pet owners, leading to mismanagement, problematic behaviour, pain, and poor health outcomes for cats.

2. What are the challenges associated with current approaches to unowned cat welfare assessment and management?

The primary management challenges identified by stakeholders include: (1) the absence of centralised, evidence-based guidelines for assessing welfare, handling, and homing unowned cats; (2) insufficient human, physical, and financial resources to address the scale of the problem; (3) limited government support for the management of unowned free-living cats; and (4) fragmented care provision, including occasional community resistance to charity-led management efforts.

3. What are the solutions and resources needed to improve the assessment and management of unowned UK cats?

Participants unanimously emphasised the need for tailored interventions that go beyond TNR and population control. Key recommendations included: (1) standardising terminology, guidelines, and procedures related to unowned cats, supported by rigorous staff training to ensure consistent management outcomes; (2) strengthening education, support, and community engagement to promote positive cat care practices and attitudes towards free-living unowned cats—including comprehensive and consistent care provision and mechanisms for reporting concerns to relevant cat welfare stakeholders [77]; (3) introducing clearer and stricter legislation around cat ownership and associated care responsibility; (4) establishing a shared responsibility framework involving coordinated efforts from relevant stakeholders such as cat owners and local community members, veterinary practices, and animal welfare charities, in addition to government engagement and financial support; and (5) strategic charity resource allocation to ensure effective balance between TNR programmes and other population management interventions, expanded volunteer recruitment, diversity and inclusion training, and the development of comprehensive databases of alternative outlets for unowned cats.

## 5. Practical Implications

Although this study focuses on the UK context, the challenges associated with the management and welfare of unowned cats are not unique to the UK [5,56], and the findings may have relevance for other countries facing similar issues. The findings of this study carry meaningful implications for those working across the shelter, policy, and veterinary sectors. For animal welfare organisations, the absence of standardised processes and welfare assessment frameworks represents a critical gap. Concerted effort is needed to develop and implement structured, evidence-based assessment tools for unowned cats, with resource allocation and management decisions grounded in proactive knowledge rather than reactive demand. Investment in comprehensive databases of alternative placement outlets and expanded volunteer networks could further strengthen the capacity to manage unowned cats effectively. For policymakers, working collaboratively with sector experts to introduce statutory guidelines around cat terminology and welfare, simplifying existing legislative language to reduce subjective interpretation, and building restorative rather than punitive frameworks, could together provide the structural foundations necessary for long-term welfare improvement. Without meaningful government engagement and financial commitment, the burden of care will continue to fall disproportionately on under-resourced charitable organisations, and the socioeconomically disadvantaged will continue to be labelled as “irresponsible” owners without the support needed to change that. For the veterinary sector, consistent protocols for assessing, triaging, and signposting unowned cats, with their welfare at the centre of decision-making, are needed; embedding these within veterinary curricula and continuing professional development frameworks could be a practical step towards achieving that.

## 6. Limitations

As a preliminary investigation, this study focused on stakeholders operating on behalf of a single UK charity. One significant limitation of this study is therefore the potentially limited scope of stakeholder perspectives collected. The study did not include stakeholders from other cat welfare organisations or rescue groups, particularly those operating on a smaller scale. This limitation raises concerns about the generalizability of the results [85], as it remains uncertain whether stakeholders from other organisations share similar views on the practices, challenges, and solutions highlighted by this study. Additionally, having all participants from the same organisation may have contributed to quicker data saturation [43] and involved potential response bias [86]. However, focusing on a single large UK cat welfare charity enabled in-depth insights to be gained across a diverse stakeholder group but within a defined operational context. It is recommended that future research should explore the perspectives of a broader range of stakeholders, to provide a more generalised and inclusive sector-wide understanding.

Another limitation pertains to the gender imbalance among the participants. While the recruited group was diverse in job roles, ages, locations, and experience within the organisation, the gender distribution was skewed, with 23 out of 25 participants identifying as female. This study adopted a constructionist epistemological framework, which considers how stakeholders’ perspectives are shaped by their social interactions with unowned cats and associated stakeholders. Gender likely affects how stakeholders work and interact with other stakeholders and how they perceive and address animal welfare issues [87,88]. However, as data on the broader gender distribution within the charity were unavailable, it is unclear whether this sample reflects the actual demographic composition of staff and volunteers or a greater willingness among females to participate. Either way, this is consistent with existing literature suggesting that caretakers of free-roaming cats tend to be predominantly female [89,90]. Future research should strive for a more even gender distribution and explore perspectives beyond the binary categories of female and male.

The recruitment strategy that relied on internal scoping and advertisement within the organisation may have inadvertently favoured more proactive or engaged stakeholders who were already well-known or willing to participate, potentially overlooking some less-engaged voices.

## Figures and Tables

**Table 1:** Themes addressing the research questions.

Research Question	Themes Addressing the Questions
RQ1: What are the primary welfare concerns for unowned cats in the UK (including those currently free-living)? RQ2: What are the challenges associated with current approaches to unowned UK cat welfare assessment and management (including those currently free-living)? RQ3: What are the solutions and resources needed for future improvements to the assessment and management of unowned UK cats (including those currently free-living)?	Theme 1: Standardisation of Terms, Guidelines, Decision-Making and Processes Theme 2: Cat Care Provision within the Community and Community Outreach Theme 3: Unowned Cat Health and Vulnerability Theme 4: Cat Ownership Theme 5: Resource Allocation and Management Theme 6: Research and Innovation

## Data Availability

The datasets presented in this article are not readily available to uphold confidential information and participant anonymity in line with data protection laws. Requests to access the datasets should be directed to avni.bhatia@cats.org.uk.

## Abbreviations

The following abbreviations are used in this manuscript:

RTA	Reflexive Thematic Analysis
ADCH	Association of Dogs and Cats Homes
TNR	Trap Neuter Return
TNVR	Trap Neuter Vaccinate Return
UoE	University of Edinburgh
FELV	Feline Leukemia Virus
FIV	Feline Immunodeficiency Virus

## Appendix A. Interview Topic Guide

Introduction:

Hello, thank you for participating in this interview. The purpose of this interview is to gather insights from various stakeholders within the charity regarding welfare concerns, management strategies, and welfare evaluation practices concerning unowned cats. In this context, ‘unowned cats’ means any cat that has not been directly relinquished to the charity from a previous owner, but which the charity provides some care for and could include stray, feral or community cats that either come directly into the charity’s care or are cared for within the community or via local veterinary practices on behalf of the charity.

Your perspectives are valuable and there is no right or wrong answer, so feel free to share your opinion with me. The interview will take approximately 60 min. If you need to take a break, please just let me know.

With your permission, I will be recording this conversation to ensure we don’t miss any important information. Your responses will be anonymised before and kept confidential.

Job role and terminology

1. Can you please start by telling me about your role within the charity and how long you have been working or volunteering with the organisation?
2. What has been your experience of working with unowned cats? [“Unowned” here is not to be explained—it is more for us to understand what the stakeholder thinks of this term and what their definition is]
3. How do you feel about some of the common terms used to describe unowned cats for example “feral”, “stray”, “street” “community” in the context of your work?
  - Are there any key characteristics or attributes of cats that fit within these categories?
  - What is your preferred terminology?
  - Are there any other terms that you commonly use to refer to unowned cats?
4. How do you feel these distinctions influence your/the charity’s approach to managing and caring for these cats?

Welfare Concerns

5. From your perspective, what are the welfare concerns for unowned cats (i.e., “feral”, “stray”, “street”, “community”, etc.) in the UK?
  - Are there specific challenges or issues that you as a stakeholder believe are particularly critical?
  - How do these concerns impact the overall welfare of the cats?
6. In an ideal world, what according to you is the place of these cats in society and what should be the ultimate outcome for these cats?
7. Ideally, should there be different solutions for the different types of unowned cats? If yes, please explain.

Welfare Management

8. What are the current strategies or programs in place within the charity that you know about for assessing and managing the welfare of unowned cats? [Highlight again, that the term “unowned” here includes interwoven concepts such as “feral”, “stray”, “street”, and “community” and can include cats that come into our care or those that do not].
  - In your opinion, how do they affect the welfare of these cats?
  - What are the strengths and weaknesses of these strategies?
9. Are there any other changes you would want to make, or suggest approaches or innovations that you believe could be effective in assessing and managing these cats’ welfare?

External interactions—stakeholders and communities

10. How important do you believe collaboration with other stakeholders (e.g., local authorities, and community organisations) is in addressing the welfare of unowned cats?
  - Probe (if needed): ask them to provide practical examples
  - What kinds of challenges or successes have we experienced in collaborating with external stakeholders?
  - Do we engage with local communities to manage unowned cats and further their welfare? If yes, what has been the response?
  - Are there any community-based initiatives or programmes that you believe have been particularly effective?

Future perspectives

11. Is there anything you would like to add before I stop recording, about the welfare of unowned cats’ welfare in the coming years?

[Emphasise that “unowned” cats include the free-living population.]

We have now reached the end of the interview.

Thank you for your valuable insights and time today. Your input will greatly contribute to our ongoing efforts. If there are any further questions or if you would like to share additional thoughts after this interview, please feel free to reach out.

## Appendix B. Theme Generation from Codes

Themes		
T1: Standardisation of Terms, Guidelines, and Decision Making	T2: Cat Care Provision within the Community and Community Outreach	T3: Unowned Cat Health and Vulnerability
Clustered Codes		
> Vets unaware about ferals > Non-awareness about cats > Lack of guidelines for community cats > More about feral cats in the veterinary curriculum > Bridging knowledge gaps within the charity > Better staff training on cat behaviour > Standardisation of terms > Ferals—negative connotations > Misinformation around terms > Misidentification of cats > Inter-organisational collaborations > Sharing of resources and knowledge > Better internal communication and education > Stringent set rules and processes in the charity > Basic standard welfare assessment tools > Focus on socialisation to decide the outcome	> Lack of responsibility; different ownership styles > Is someone looking out for the cat > More public education > Focus on human behaviour change models; encourage more responsibility > Collaboration with community, local leaders > Helping caregivers of community cats > Partial care/semi-ownership > Proactive not reactive approaches > More open conversations around euthanasia	> Human abuse/mistreatment > Seen as pests for nuisance behaviour > Road accidents, injuries > Unsafe environment > No constant food, water, or shelter source > Not being neutered, overpopulation > FIV, FeLV diseases; other diseases > Lack of basic vet care > Exposure to parasites > Malnourished/overweight cats > Hypothermia > Kitten mortality > Excess fights, competition > Wounds and abscesses
Themes		
T4: Cat Ownership	T5: Resource Allocation and Management	T6: Research and Innovation
Clustered Codes		
> Focus on human behaviour change models; encourage more responsibility > Microchip legislation > Stricter laws > Government funding and intervention > Supporting and educating cat owners	> Neutering community cats > Neutering domestic cats > More resources—money, staff, space > Hire paid TNR staff > Hire more volunteers > More diversity training and inclusion > Dedicated team for unowned cats > Database of specialist fosterers for kittens > Database of suitable outlets for outdoorsy cats > Database of outlets for disabled cats	> More research > Designing special pens/cages > Ongoing monitoring of interventions > Follow-up documentation

## Appendix C. Participant Demographics

**Participant ID**	**Main Type of Cat Care Activities Involved In**	**Number of Years in Organisation**	**Position**	**Region of** **Work**	**Age**	**Gender** **(F/M)**
P1	Adoption	15 Years	Staff	Urban	41	M
P2	Adoption	5.5 Years	Staff	Urban	29	F
P3	Neutering	13 Years	Volunteer turned to Staff	Urban	39	F
P4	Volunteering	45 Years	Volunteer	Urban	62	F
P5	Welfare and Homing	10 Years	Staff	Urban	50	M
P6	Community Engagement	2 Years	Staff	Urban	29	F
P7	Veterinary	2 Years	Staff	Urban	40	F
P8	Branch Coordination	16 Years	Volunteer	Rural	66	F
P9	Community Engagement	5 Years	Staff	Rural	48	F
P10	Rehoming and Welfare	1.5 Years	Volunteer turned to Staff	Urban	24	F
P11	Regional Cat Welfare	3 Years	Staff	Urban	32	F
P12	Regional Cat Welfare	10 Years	Staff	Rural	38	F
P13	Branch Coordination, Neutering	20 Years	Volunteer	Rural	67	F
P14	Rehoming & Welfare	13 Years	Volunteer turned to Staff	Urban	41	F
P15	Adoption	6 Years	Staff	Rural	35	F
P16	Neutering	1.5 Years	Volunteer	Urban	37	F
P17	Feline Behaviour	14 Years	Staff	Rural	40	F
P18	Volunteer	5 Years	Volunteer	Rural	33	F
P19	Volunteering	16 Years	Volunteer turned to Staff	Urban	47	F
P20	Operations	5 Years	Volunteer turned to Staff	Rural	44	F
P21	Projects	11 Years	Volunteer turned to Staff	Urban	65	F
P22	Branch Coordination	4 Years	Staff	Urban	46	F
P23	Community Engagement	34 Years	Volunteer turned to Staff	Rural	61	F
P24	Cat Welfare	2.5 Years	Volunteer	Urban	44	F
P25	Neutering	5 Years	Staff	Urban	50	F
